# GLRB variants regulate nearby gene expression in human brain tissues

**DOI:** 10.1038/s41598-017-13702-8

**Published:** 2017-10-17

**Authors:** Qing-jian Wu, Ming-feng Yang, Pi-da Hao, Cheng-jun Yan, Chun-jing Du, Han-xia Li, Ya-jun Hou, Bao-liang Sun, Shu-yin Sun

**Affiliations:** 1 Department of Emergency, Jining NO.1 People’s Hospital, Jining, Shandong 272011 China; 2 Department of Neurology, Key Laboratory of Cerebral Microcirculation in Universities of Shandong, Affiliated Hospital of Taishan Medical University, Taian, Shandong 271000 China; 3 Department of Neurology, Shandong University School of Medicine, Jinan, Shandong 250012 China; 4 Department of neurology, Shan xian Dong Da Hospital, Shanxian, Heze, Shandong 274300 China

## Abstract

A recent genome-wide association study (GWAS) identified four genetic variants rs78726293, rs191260602, rs17035816 and rs7688285 in GLRB gene to be associated with panic disorder (PD) risk. In fact, GWAS is an important first step to investigate the genetics of human complex diseases. In order to translate into opportunities for new diagnostics and therapies, we must identify the genes perturbed by these four variants, and understand how these variant functionally contributes to the underlying disease pathogenesis. Here, we investigated the effect of these four genetic variants and the expression of three nearby genes including PDGFC, GLRB and GRIA2 in human brain tissues using the GTEx (version 6) and Braineac eQTLs datasets. In GTEx (version 6) dataset, the results showed that both rs17035816 and rs7688285 variants could significantly regulate PDGFC and GLRB gene expression. In Braineac dataset, the results showed that rs17035816 variant could significantly regulate GLRB and GRIA2 gene expression. We believe that these findings further provide important supplementary information about the regulating mechanisms of rs17035816 and rs7688285 variants in PD risk.

## Introduction

Panic disorder (PD) is a kind of anxiety disorder, which is prevalent in a 2–3% life-time, and could cause a huge burden of disease^1^. Deckert *et al*. recently performed a genome-wide association study of PD/agoraphobia (AG) using large-scale sample size^1^. They successfully identified four genetic variants rs78726293, rs191260602, rs17035816 and rs7688285 in GLRB gene^1^. They further conducted an expression quantitative trait loci (eQTL) analysis to detect the functional effect of these four variants on the expression of GLRB and neighbor genes using the Genotype-Tissue Expression (GTEx) eQTL database^1^. Meanwhile, they evaluated the potential association between rs7688285 and GLRB mRNA expression levels using the post-mortem brain samples of 76 individuals^1^. Their results showed that none of these four genetic variants could regulate the expression of nearby genes in the GTEx database^1^. In post-mortem brain samples, Deckert *et al*. reported significant association between rs7688285 A allele and the increased mean expression of GLRB with beta = 0.498 and *P* = 0.013 in the midbrain, but not in the forebrain with *P* = 0.421 or in the amygdalae with *P* = 0.487^1^.

Deckert *et al*. selected the online GTEx eQTL database to evaluate the potential association between these four genetic variants and gene expression of their nearby genes^1^. In fact, the online GTEx eQTL database only included the all significant variant-gene pairs with a genome wide false discovery rate (FDR) threshold of 0.05^2^. The suggested association between these four genetic variants and gene expression of their nearby genes may have been adjusted by the genome wide FDR threshold, and may not be included in the online GTEx eQTL database. It is important to evaluate these findings using all the original SNP-gene associations in GTEx database.

Meanwhile, evidence shows that the effect of genetic variants on gene expression may occur in disease-relevant tissue types^3–9^. It is still necessary to investigate these potential expression associations in other human brain tissues, as Deckert *et al*. did using the human brain samples. Here, we investigated the effect of these four genetic variants rs78726293, rs191260602, rs17035816 and rs7688285 and the expression of three nearby genes including PDGFC, GLRB and GRIA2 using two eQTLs datasets.

## Results

### eQTLs analysis in GTEx dataset

In GTEx (version 6) dataset, we found that rs78726293 and rs191260602 were not available in the. We then focused on the two genetic variants including rs17035816 and rs7688285 and three genes including PDGFC, GLRB and GRIA2 in the following analysis. The results indicated that both rs17035816 and rs7688285 variants could significantly regulate nearby gene expression in human brain tissues (significance threshold 0.05)^10^. In brief, rs17035816 variant could significantly regulate PDGFC gene expression in cerebellar hemisphere tissue (*P* = 8.70E-03), putamen basal ganglia tissue (*P* = 4.14E-03) and cerebellum tissue (*P* = 3.17E-02), and regulate GLRB gene expression in cerebellar hemisphere tissue (*P* = 1.50E-03). The rs7688285 variant could significantly regulate PDGFC gene expression in hippocampus tissue (*P* = 2.03E-02) and putamen basal ganglia tissue (*P* = 1.37E-02), and regulate GLRB gene expression in hypothalamus tissue (*P* = 2.63E-02). We further performed a multiple testing correction using a FDR threshold of 0.05 in these 10 brain tissues. Interestingly, rs17035816 variant still significantly regulates nearby gene expression after the multiple hypothesis test correction. More detailed results are described in Table 1.

**Table 1:** rs7688285 and rs17035816 variants and nearby gene expression in GTEx.

SNP	Effect Allele	Beta	*P* value	FDR	Gene	Sample size	Tissue
rs7688285	A	0.106	0.23	0.64	PDGFC	72	Anterior cingulate cortex (BA24)
rs7688285	A	0.090	0.48	0.64	PDGFC	100	Caudate (basal ganglia)
rs7688285	A	−0.100	0.58	0.64	PDGFC	89	Cerebellar Hemisphere
rs7688285	A	0.106	0.52	0.64	PDGFC	103	Cerebellum
rs7688285	A	−0.094	0.47	0.64	PDGFC	96	Cortex
rs7688285	A	−0.093	0.52	0.64	PDGFC	92	Frontal Cortex (BA9)
rs7688285*	**A**	**0**.**296**	**2**.**03E-02**	**0**.**10**	**PDGFC**	**81**	**Hippocampus**
rs7688285	A	0.138	0.39	0.64	PDGFC	81	Hypothalamus
rs7688285	A	−0.024	0.86	0.86	PDGFC	93	Nucleus accumbens (basal ganglia)
**rs7688285**	**A**	**−0**.**297**	**1**.**37E-02**	**0**.**10**	**PDGFC**	**82**	**Putamen** (**basal ganglia**)
rs7688285	A	0.036	0.42	0.66	GLRB	72	Anterior cingulate cortex (BA24)
rs7688285	A	0.133	0.05	0.26	GLRB	100	Caudate (basal ganglia)
rs7688285	A	−0.091	0.46	0.66	GLRB	89	Cerebellar Hemisphere
rs7688285	A	−0.034	0.76	0.92	GLRB	103	Cerebellum
rs7688285	A	0.001	0.99	0.99	GLRB	96	Cortex
rs7688285	A	0.014	0.83	0.92	GLRB	92	Frontal Cortex (BA9)
rs7688285	A	0.081	0.25	0.66	GLRB	81	Hippocampus
**rs7688285**	**A**	**0**.**192**	**2**.**63E-02**	**0**.**26**	**GLRB**	**81**	**Hypothalamus**
rs7688285	A	0.079	0.28	0.66	GLRB	93	Nucleus accumbens (basal ganglia)
rs7688285	A	0.067	0.37	0.66	GLRB	82	Putamen (basal ganglia)
rs17035816	G	0.104	0.46	0.55	PDGFC	72	Anterior cingulate cortex (BA24)
rs17035816	G	−0.099	0.46	0.55	PDGFC	100	Caudate (basal ganglia)
**rs17035816**	**G**	**0**.**449**	**8**.**70E-03**	**4**.**35E-02**	**PDGFC**	**89**	**Cerebellar Hemisphere**
**rs17035816**	**G**	**0**.**402**	**3**.**17E-02**	**0**.**11**	**PDGFC**	**103**	**Cerebellum**
rs17035816	G	0.153	0.28	0.55	PDGFC	96	Cortex
rs17035816	G	0.232	0.09	0.22	PDGFC	92	Frontal Cortex (BA9)
rs17035816	G	0.115	0.50	0.55	PDGFC	81	Hippocampus
rs17035816	G	0.165	0.45	0.55	PDGFC	81	Hypothalamus
rs17035816	G	−0.092	0.56	0.56	PDGFC	93	Nucleus accumbens (basal ganglia)
**rs17035816**	**G**	**0**.**361**	**4**.**14E-03**	**4**.**14E-02**	**PDGFC**	**82**	**Putamen** (**basal ganglia**)
rs17035816	G	−0.047	0.50	0.68	GLRB	72	Anterior cingulate cortex (BA24)
rs17035816	G	0.038	0.61	0.68	GLRB	100	Caudate (basal ganglia)
**rs17035816**	**G**	**0**.**371**	**1**.**50E-03**	**1**.**50E-02**	**GLRB**	**89**	**Cerebellar Hemisphere**
rs17035816	G	0.089	0.48	0.68	GLRB	103	Cerebellum
rs17035816	G	0.011	0.89	0.89	GLRB	96	Cortex
rs17035816	G	−0.031	0.61	0.68	GLRB	92	Frontal Cortex (BA9)
rs17035816	G	−0.064	0.49	0.68	GLRB	81	Hippocampus
rs17035816	G	−0.113	0.35	0.68	GLRB	81	Hypothalamus
rs17035816	G	−0.122	0.15	0.68	GLRB	93	Nucleus accumbens (basal ganglia)
rs17035816	G	−0.091	0.24	0.68	GLRB	82	Putamen (basal ganglia)

*Significant associations (*P* < 0.05) are bolded. rs7688285, chr4:157968618, A/G; rs17035816, chr4:158088464, A/G; Significance level for a potential association is 0.05; Beta is the regression coefficient based on the effect allele. Beta > 0 and Beta < 0 means that this effect allele regulates increased and reduced gene expression, respectively.

### eQTLs analysis in Braineac dataset

In Braineac dataset, we found that rs78726293, rs191260602 and rs7688285 were not available. We then focused on the rs17035816 variant and three genes including PDGFC, GLRB and GRIA2 in the following analysis. The results showed that rs17035816 variant could significantly regulate nearby gene expression in human brain tissues (significance threshold 0.05). In brief, rs17035816 variant could significantly regulate GLRB and GRIA2 gene expression in cerebellar cortex tissue with *P* = 1.49E-02 and *P* = 3.49E-02, respectively. More detailed results are described in Table 2.

**Table 2:** rs17035816 variant and gene expression in Braineac.

SNP	Effect Allele	Beta	*P* value	Gene	Brain Tissues	ID
**rs17035816**	**G**	**0**.**256**	**1**.**49E-02**	**GLRB**	**Cerebellar cortex**	**t2749191**
rs17035816	G	0.161	2.48E-01	GLRB	Frontal cortex	t2749191
rs17035816	G	0.109	4.36E-01	GLRB	Hippocampus	t2749191
rs17035816	G	0.108	4.78E-01	GLRB	Medulla	t2749191
rs17035816	G	0.042	8.14E-01	GLRB	Occipital cortex	t2749191
rs17035816	G	−0.01	9.48E-01	GLRB	Putamen	t2749191
rs17035816	G	0.346	6.37E-02	GLRB	Substantia nigra	t2749191
rs17035816	G	0.062	6.92E-01	GLRB	Temporal cortex	t2749191
rs17035816	G	−0.047	8.15E-01	GLRB	Thalamus	t2749191
rs17035816	G	−0.04	7.44E-01	GLRB	Intralobular white matter	t2749191
**rs17035816**	**G**	**0**.**143**	**3**.**79E-02**	**GRIA2**	**Cerebellar cortex**	**t2749222**
rs17035816	G	0.068	4.81E-01	GRIA2	Frontal cortex	t2749222
rs17035816	G	0.019	8.63E-01	GRIA2	Hippocampus	t2749222
rs17035816	G	0.067	5.48E-01	GRIA2	Medulla	t2749222
rs17035816	G	0	9.98E-01	GRIA2	Occipital cortex	t2749222
rs17035816	G	−0.007	9.61E-01	GRIA2	Putamen	t2749222
rs17035816	G	0.176	2.84E-01	GRIA2	Substantia nigra	t2749222
rs17035816	G	0.057	6.29E-01	GRIA2	Temporal cortex	t2749222
rs17035816	G	−0.026	8.27E-01	GRIA2	Thalamus	t2749222
rs17035816	G	−0.105	3.21E-01	GRIA2	Intralobular white matter	t2749222
rs17035816	G	−0.109	7.55E-02	PDGFC	Cerebellar cortex	t2791197
rs17035816	G	0.008	9.01E-01	PDGFC	Frontal cortex	t2791197
rs17035816	G	−0.005	9.42E-01	PDGFC	Hippocampus	t2791197
rs17035816	G	−0.078	2.15E-01	PDGFC	Medulla	t2791197
rs17035816	G	−0.013	8.15E-01	PDGFC	Occipital cortex	t2791197
rs17035816	G	−0.072	3.47E-01	PDGFC	Putamen	t2791197
rs17035816	G	−0.046	5.00E-01	PDGFC	Substantia nigra	t2791197
rs17035816	G	0.039	5.89E-01	PDGFC	Temporal cortex	t2791197
rs17035816	G	−0.002	9.75E-01	PDGFC	Thalamus	t2791197
rs17035816	G	−0.05	4.23E-01	PDGFC	Intralobular white matter	t2791197

*Significant associations (*P* < 0.05) are bolded. rs17035816, chr4:158088464, A/G; Significance level for a potential association is 0.05; Beta is the regression coefficient based on the effect allele. Beta > 0 and Beta < 0 means that this effect allele regulates increased and reduced gene expression, respectively.

### Meta-analysis

We further performed a meta-analysis in the same brain tissues including Cerebellum, Hippocampus, Frontal cortex, and Putamen. The results showed that rs17035816 variant could significantly regulate GLRB gene expression in cerebellum tissue. More detailed results are described in Table 3.

**Table 3:** Meta-analysis of GTEx and Braineac datasets in four brain tissues.

SNP	Effect Allele	GTEx		Braineac		Meta		Tissue	Gene
		Beta	SE	Beta	SE	Beta	*P*		
rs17035816	G	0.402	0.184	−0.109	0.061	0.117	0.645	Cerebellum	PDGFC
rs17035816	G	0.232	0.134	0.008	0.063	0.089	0.410	Frontal Cortex	PDGFC
rs17035816	G	0.115	0.170	−0.005	0.063	0.009	0.874	Hippocampus	PDGFC
rs17035816	G	0.361	0.121	−0.072	0.077	0.135	0.535	Putamen	PDGFC
**rs17035816**	**G**	**0**.**089**	**0**.**125**	**0**.**256**	**0**.**104**	0.188	**0**.**019**	**Cerebellum**	**GLRB**
rs17035816	G	−0.031	0.061	0.161	0.139	0.000	0.995	Frontal Cortex (BA9)	GLRB
rs17035816	G	−0.064	0.093	0.109	0.140	−0.011	0.885	Hippocampus	GLRB
rs17035816	G	−0.091	0.078	−0.010	0.155	−0.075	0.281	Putamen (basal ganglia)	GLRB

rs17035816, chr4:158088464, A/G; Significance level for a potential association is 0.05; Beta is the regression coefficient based on the effect allele. Beta > 0 and Beta < 0 means that this effect allele regulates increased and reduced gene expression, respectively. SE, standard error.

## Discussion

Deckert *et al*. highlighted four genetic variants rs78726293, rs191260602, rs17035816 and rs7688285 in GLRB gene to be associated with PD risk^1^. In fact, GWAS is an important first step to investigate the genetics of human complex diseases as widely described in previous studies^11–25^. In order to translate into opportunities for new diagnostics and therapies, we must identify the genes perturbed by these four variants, and understand how these variant functionally contributes to the underlying disease pathogenesis^3–8,12,26–30^. If a genetic variant is associated with increased or decreased expression of a particular gene, this suggests that the gene on which the variant acts could be in the causal pathway^31^. However, Deckert *et al*. revealed no significant cis-eQTL using the online GTEx database^1^. Here, we successfully identified significant cis-eQTL using all the original SNP-gene summary association results in the GTEx (version 6), even after the multiple hypothesis test correction using FDR threshold of 0.05.

In the GTEx dataset, we confirmed previous findings. Deckert *et al*. analyzed the post-mortem brain samples of 76 individuals, and identified that the rs7688285 A allele could significantly regulate increased mean expression of GLRB with beta = 0.498 and *P* = 0.013^1^. Here, our findings showed that rs7688285 variant A allele could significantly regulate increased PDGFC gene expression in hippocampus tissue (beta = 0.296 and *P* = 2.03E-02), reduced PDGFC gene expression in putamen basal ganglia tissue (beta = −0.297 and *P* = 1.37E-02), and increased GLRB gene expression in hypothalamus tissue (beta = 0.192 and *P* = 2.63E-02). Meanwhile, the results also showed some novel findings. Take rs17035816 variant for example, it could significantly regulate nearby gene expression even after the multiple hypothesis test correction as described in Table 1.

We further evaluated the potential association between these four genetic variants and the expression of three nearby genes including PDGFC, GLRB and GRIA2 in the Braineac dataset including 10 brain regions from 134 neuropathologically normal individuals of European descent^32^. Interestingly, the rs17035816 could significantly regulate increased GLRB and GRIA2 gene expression in cerebellar cortex. We believe that these findings further provide important supplementary information about the regulating mechanisms of rs17035816 and rs7688285 variants in PD risk in the human brain tissues. Genetic variants may need tissue, cell, region, disease specific factors to exert their influences on gene expression^33,34^. Here, we identified different results in GTEx (version 6) and Braineac eQTLs datasets. We think that disease status may influence the association between these genetic variants and GLRB and GRIA2 gene expression.

Here, the two variants rs78726293 and rs191260602 are not available in the GTEx dataset. Three variants rs78726293, rs191260602, and rs7688285 are not available in the Braineac dataset. We have used HaploReg (version 4) to identify the proxy SNPs based on the linkage disequilibrium (LD) information in 1000 Genomes Project (EUR) with r^2^ ≥ 0.8^2^. However all these tagged SNPs are still not available in the GTEx dataset and the Braineac dataset. We think that following studies should further evaluate these potential expression associations using other eQTLs datasets in human brain regions.

## Materials and Methods

### The GTEx dataset

The GTEx (version 6) eQTLs dataset included 53 tissues, 544 donors and 8555 samples^35^. These 544 donors have several death pathologies including traumatic injury, cerebrovascular disease, heart disease, liver, renal, respiratory, and neurological diseases^35^. Here, we selected 10 human brain tissues including anterior cingulate cortex, caudate basal ganglia, cerebellar hemisphere, cerebellum, cortex, frontal cortex BA9, hippocampus, hypothalamus, nucleus accumbens basal ganglia, and putamen basal ganglia, which include at least 70 samples^10^. The GTEx used the RNA-Seq method to measure the gene expression^10^.

### The Braineac dataset

The Braineac eQTLs dataset is from a web server for data from the UK Brain Expression Consortium (UKBEC)^32^. This dataset includes 10 brain regions from 134 neuropathologically normal individuals of European descent^32^. The 10 brain regions are cerebellar cortex, frontal cortex, hippocampus, medulla, occipital cortex, putamen, substantia nigra, temporal cortex, thalamus, and intralobular white matter^32^. The Braineac used the Affymetrix GeneChip Human exon 1.0 ST arrays to measure the gene expression^32^. The gene expression in transcript level is the Winsorised mean over exon-specific levels^32^.

### eQTLs analysis

In the GTEx dataset, a linear regression analysis was applied to evaluate the SNP-gene expression association using R package Matrix eQTL, assuming an additive model and adjusting for the covariates including genotyping PCs, genotyping array platform, 15, 30 or 35 PEER factors, and the gender^10^. Here, we downloaded all the SNP-gene association summary results from the GTEx (version 6) database to directly evaluate the potential association between these four genetic variants and gene expression of nearby genes. In the Braineac dataset, a linear regression analysis was also applied to evaluate the potential association between genetic variants and the expression of nearby genes using the R package Matrix EQTL^32^. Here, we downloaded the gene expression data and the genotype data of genetic variants with 1 Mb upstream of transcription start site and 1 Mb downstream of transcription end site from the Braineac online database^32^. We then evaluated the potential SNP-gene expression association using the R program. More detailed information is described in a recent study^36^.

### Meta-analysis

In the same brain tissue, we performed a meta-analysis to increase the statistical power. The pooled effect was calculated using the fixed effect model (Mantel-Haenszel) or the random-effect model (DerSimonian-Laird) determined by the heterogeneity^3–8,12,26–30^. A Z test was used to determine the significance of the effect. All tests were computed using the R Package (http://cran.r-project.org/web/packages/meta/index.html).
